# Thromboelastography (TEG) parameters as potential predictors of malignancy and tumor progression in colorectal cancer

**DOI:** 10.1186/s12957-023-03237-w

**Published:** 2023-11-17

**Authors:** Zhang-Sheng Zhao, Yang-Cong Qi, Jing-Wei Wu, Li-Hui Qian, Bin Hu, You-Li Ma

**Affiliations:** 1 Department of Blood Transfusion, Ningbo Medical Treatment Center Lihuili Hospital, Ningbo, Zhejiang People’s Republic of China; 2 Department of Clinical Laboratory, Ningbo Medical Treatment Center Lihuili Hospital, Ningbo, Zhejiang People’s Republic of China

**Keywords:** Thromboelastography, Colorectal cancer, Hypercoagulability, Diagnosis, Conventional laboratory parameters

## Abstract

**Purpose:**

The purpose of this study was to investigate the use of thromboelastography (TEG) in patients with colorectal cancer and to examine whether the TEG parameters can be used as potential markers for disease screening and prediction of disease severity.

**Methods:**

One-hundred fifteen healthy controls (HC), 43 patients with benign adenoma (BA), and 387 patients with colorectal cancers (CRC) were included in the study. TEG parameters (reaction time, R; clot kinetics, K; alpha angle, α-angle; maximum amplitude, MA), conventional laboratory parameters, and clinical information were collected and analyzed among the HC, BA, and CRC groups. Receiver operating characteristics (ROC) were used for differential analysis. The correlation between TEG parameters and pathological information of CRC (differentiation degree, vaso-nerve infiltration, TNM stage) was analyzed. The differences in TEG parameters at different stages of disease and pre-/post operation were compared.

**Results:**

Shorter K and higher α-angle/MA were found in patients with CRC compared with HC and BA (*P* < 0.001). TEG parameters demonstrated moderate diagnostic value (distinguish CRC from HC + BA: *K-AUC* = 0.693, α-angle-AUC = 0.687, *MA-AUC* = 0.700) in CRC but did not outperform traditional laboratory parameters. TEG hypercoagulability was closely associated with tumor markers (carcinoma embryonic antigen and carbohydrate antigen 19–9) and pathological information (differentiation degree, vaso-nerve infiltration, and TNM stage) (*P* < 0.05). Trend analysis showed that K decreased, but α-angle/MA increased gradually as the tumor progressed (*P* < 0.001). K- and α-angle showed slightly better sensitivity in predicting advanced tumors compared to traditional laboratory parameters. In CRC patients, 3–6 months after tumor resection, K [from 1.8 (1.5, 2.3) to 1.9 (1.6, 2.6)], α-angle [from 65.3 (59.0, 68.6) to 63.7 (56.6, 68.5)], and MA [from 61.0 (58.2, 66.0) to 58.9 (55.8, 61.3)] exhibited modest improvements compared to their preoperative values (*P* < 0.05).

**Conclusion:**

TEG parameters possess moderate diagnostic value in CRC diagnosis and predicting advanced tumors, and they are closely linked to surgical interventions. Although TEG parameters do not significantly outperform traditional laboratory parameters, they still hold promise as potential alternative indicators in CRC patients.

## Introduction

The incidence and mortality of colorectal cancer (CRC) have been vastly growing in China during the last decade, and it was estimated that there were 376,300 new onsets and 191,000 deaths in 2015 [1]. Although the time span for benign adenoma (BA) to develop into CRC takes many years, the prognosis is poor once the tumor metastasizes to lymph nodes or distant organs [2]. Thus, great emphasis should be placed on the identification of CRC from benign adenoma, especially in the differential diagnosis of advanced CRC.

Plenty of evidence has proved that malignancy was usually accompanied by abnormal thrombosis, characterized by activation of clotting factors, elevated fibrinogen, and high platelet responsiveness. Malignancy-associated coagulopathy is considered to play an essential role in tumor onsets and metastasis by manifold strategies, so identifying markers of this coagulopathy can be used as potential indicators for the staging of tumor progression. Conventional coagulation tests (CCTs), such as fibrinogen, prothrombin time (PT), activated partial thromboplastin time (APTT), and thrombin time (TT), have traditionally been used to identify malignancy-associated coagulopathy. However, CCTs have their limitations because the methods are based on plasma analysis and only reflect static snapshots of coagulation properties [3, 4].

Thromboelastography (TEG) is a method that graphs and evaluates the clot’s strength and elasticity from the initiation of clotting factors to fibrinolysis [5], providing a more comprehensive coagulation information status than the conventional coagulation tests (CCT). Accordingly, TEG was widely used in surgery and intensive care units to monitor abnormal coagulopathy and guide transfusion [6, 7]. Recently, TEG has been largely applied to the identification of hypercoagulable states of solid tumors such as liver cancer [8], lung cancer [9], pancreatic cancer [10], and other tumors, among which TEG was more sensitive than CCTs in recognition of abnormal coagulation status in prostate cancer [11] and kidney tumor [12]. Besides, many studies have found that TEG had the potential to identify malignant tumors [13] and even correlated with disease progression [14]. However, few data have been reported on the clinical use of TEG parameters in CRC patients.

Therefore, we conducted a retrospective analysis of preoperative TEG parameters in health controls (HC), patients with colorectal benign adenoma (BA), and patients with colorectal cancer (CRC) to explore whether they can identify malignant tumors and predict the stage of tumor progression.

## Methods

### Study setting

This was a retrospective study involving 687 health controls (HC), patients with benign adenoma (BA), and patients with colorectal cancer (CRC) treated at Ningbo Medical Treatment Center Lihuili Hospital of Zhejiang Province from January 2019 to October 2022. Patient data, including age, gender, lifestyles (smoking and drinking history), previous medical history (hypertension, hyperlipidemia, diabetes, and coronary heart disease), traditional laboratory parameters (hemoglobin, platelet, CCTs), tumor markers (carcinoma embryonic antigen, CEA; carbohydrate antigen 19–9, CA19-9), and pathological information (differentiation degree, vaso-nerve infiltration, TNM stage, etc.), were documented from the electronic medical record system. The cancer stage was determined according to the 7th TNM staging system by the American Joint Committee for Cancer (AJCC) [15]. Given the nature of the retrospective analysis, we requested an exemption from informed consent, and the study was approved by the Ningbo Medical Center Lihuili Hospital Ethics Committee (approval no. KY2023SL126-01).

Inclusion criteria were as follows: (1) HC: individuals who passed the annual physical examination in our hospital and were confirmed to have no intestinal diseases (get a colonoscopy); (2) BA: patients with intestinal space occupation underwent endoscopic mass resection and were pathologically identified as benign adenoma; and (3) CRC: patients diagnosed with colorectal cancer and underwent surgical resection in our hospital. Exclusion criteria were as follows: (1) Having other types of malignancies; (2) having rheumatic immune diseases; (3) taking anticoagulants or anti-inflammatory drugs within 2 weeks; (4) suffering acute bleeding or cardiovascular/cerebrovascular obstruction; (5) severe infection within 2 weeks, recently transfused with any blood products and preoperative adjuvant radiotherapy or chemotherapy; and (6) TEG data are not available.

### Thromboelastography (TEG) measurement

The Thromboelastograph®5000 Hemostasis System was carried out to test TEG parameters. Briefly, 340 μl of sodium citrate anticoagulant whole blood and kaolin mixture was placed into a disposable TEG cup, and then, 20 μl of 0.2-M CaCl_2_ was added to start coagulation. The torque generated by blood coagulation was converted into an electrical signal by the torsion wire, which was recorded in the software to form the clotting parameters. The meaning of TEG parameters has been discussed in our previous study [16], and the brief interpretation and the normal range of each parameter are as follows: reaction time (R, normal range: 5.0–10.0 min) represents the time of thrombin generation and is an indicator of activation of clotting factors; clot kinetics (K, normal range: 1.0–3.0 min) represents the time required for fibrin activation and is an indicator of fibrinogen function; alpha angle (α-angle, normal range: 53.0–72.0 deg) represents the speed of clot formation and is another indicator of fibrinogen function; and maximum amplitude (MA, normal range: 50.0–70.0 mm) represents the maximum strength of the clot and is an indicator of both platelet function (approximately 80%) and fibrinogen function (approximately 20%) [17]. According to the instrument’s instructions, hypercoagulability was defined as if TEG parameters met one of the conditions: *R* < 5.0 min; *K* < 1.0 min; α-angle > 72.0 deg; and *MA* > 70.0 mm. Thus, we divided TEG parameters into low R group (LR, *R* < 5.0 min) and high R group (HR, *R* ≥ 5.0 min); low K group (LK, *K* < 1.0 min) and high K group (HK, *K* ≥ 1.0 min); low α-angle group (Lα, α-angle ≤ 72.0 deg) and high α-angle group (Hα, α-angle > 72.0 deg); and low MA group (LMA, *MA* ≤ 70.0 mm) and high MA group (LMA, *MA* > 70.0 mm).

### Statistical analyses

SPSS software (IBM, 26.0, USA) and GraphPad Prism software (GraphPad, 8.0, USA) were performed for statistical analyses. Due to some variables were not normally distributed after being tested by the Kolmogorov–Smirnov test, all continuous variables were represented as the median (interquartile range). Categorical data were represented as the number (percentage, %). Propensity score matching (PSM) was used to reduce the bias of age, sex, lifestyle, and medical history (match tolerance set to 0.02). Kruskal–Wallis test, chi-square test, Mann–Whitney *U*-test, and paired-samples *T*-test were used to compare the differences between groups. When it comes to pairwise comparisons, *P*-value correction was performed by Bonferroni correction. The Jonckheere-Terpstra test was used to test the one-way tendency of TEG parameters. The receiver operating characteristic (ROC) analysis was performed to evaluate the identifying potential of TEG parameters in CRC with the Youden index as the optimal critical value. *P* < 0.05 was considered statistically significant (two sided).

## Results

To accurately reflect the effect of colorectal tumor on TEG parameters, we applied a detailed data screening. A total of 142 participants were excluded from the data screening for the following reasons: Having other types of malignancies (*n* = 10), having rheumatic immune disease (*n* = 5), taking anticoagulants or anti-inflammatory drugs (*n* = 10), acute bleeding or obstruction (*n* = 7), transfusion (*n* = 11), infection (*n* = 15), preoperative adjuvant chemotherapy or radiotherapy (*n* = 17); no chance for surgery resection (*n* = 30), and TEG data are not available (*n* = 37). Finally, 115 HC, 43 patients with BA, and 387 patients with CRC were included after data exclusion. The primary demographic and clinical characteristics of the included participants were shown in Table 1. In brief, when compared with the HC group, the CRC group had higher levels of age, male ratio, smoking/drinking rate, PT, fibrinogen, D-dimer, CEA, and CA19-9 (*P* < 0.05). However, lower levels of hemoglobin and APTT were observed in the CRC group compared with the HC group (*P* < 0.05). In comparison with the BA group, the CRC group also manifested lower hemoglobin, higher platelet, higher fibrinogen, and higher CEA (*P* < 0.05). The significant difference between the HC group and the BA group was only displayed in hemoglobin and D-dimer (*P* < 0.05).

**Table 1:** Characteristics of healthy controls (HC), benign adenoma (BA) patients, and colorectal cancer (CRC) patients

	Before matching							After matching		
Variable	HC	*P*^a^	BA	*P*^b^	(CRC)	*P*^c^	*P*^d^	HC + BA	CRC	*P*^e^
No. of cases	115	/	43	/	387	/	/	140	140	/
Age (years)	59.0 (50.0, 65.0)	0.077^*^	62.0 (55.0, 69.0)	0.206^*^	65.0 (59.0, 72.0)	< 0.001^*^	< 0.001^*^	62 (55.68)	61 (52.67)	0.277^^^
Sex (male) (*n*, %)	53 (46.1)	0.828^^^	24 (55.8)	1.000^^^	232 (59.9)	**0.025**^^^	**0.031**^^^	77 (55.0)	76 (54.3)	0.904^^^
Life style										
Smoking (*n*, %)	10 (8.7)	0.513^^^	7 (16.3)	1.000^^^	80 (20.7)	**0.010**^^^	**0.012**^^^	17 (12.1)	19 (13.6)	0.721^^^
Drinking (*n*, %)	10 (8.7)	0.989^^^	6 (14.0)	1.000^^^	67 (17.3)	**0.073**^^^	0.076^^^	16 (11.4)	19 (13.6)	0.588^^^
Medical history										
Hypertension (*n*, %)	/	/	19 (44.2)	/	146 (37.7)	/	0.409^^^	19 (13.6)	16 (11.4)	0.588^^^
Hyperlipidemia (*n*, %)	/	/	12 (27.9)	/	49 (12.7)	/	**0.007**^^^	12 (8.6)	7 (5.0)	0.235^^^
Diabetes (*n*, %)	/	/	6 (14.0)	/	48 (12.4)	/	0.771^^^	6 (4.3)	6 (4.3)	1.000^^^
Coronary heart disease (*n*, %)	/	/	0 (0.0)	/	10 (2.6)	/	0.608^^^	0 (0)	0 (0)	/
Laboratory parameters										
Hb (g/L)	141.0 (132.0, 150.0)	0.032^*^	133.0 (124.0, 145.0)	0.007^*^	126.0 (108.0, 137.0)	< 0.001^*^	< 0.001^*^	139.5 (130.0, 149.0)	125.0 (107.5, 136.0)	**< 0.001**^#^
PLT (× 10^9^/L)	209.5 (190.0, 237.0)	0.932^*^	206.5 (153.0, 236.0)	0.022^*^	224.0 (183.0, 272.0)	0.054^*^	0.004^*^	208.5 (181.0, 236.0)	229.0 (187.0, 276.5)	**0.001**^#^
PT (s)	11.4 (11.1, 11.9)	1.000^*^	11.5 (11.1, 11.9)	0.052^*^	11.8 (11.3, 12.4)	< 0.001^*^	< 0.001^*^	11.4 (11.1, 11.9)	12.0 (11.4, 12.6)	**< 0.001**^#^
APTT (s)	31.4 (29.5, 32.9)	1.000^*^	30.6 (28.7, 32.8)	0.731^*^	30.3 (28.6, 32.3)	0.002^*^	0.003^*^	31.3 (29.1 32.9)	30.2 (29.0 32.3)	0.051^#^
TT (s)	14.4 (13.7, 15.0)	1.000^*^	14.9 (13.9, 15.4)	1.000^*^	14.4 (13.5, 15.3)	1.000^*^	0.420^*^	14.5 (13.7, 15.1)	14.5 (13.8, 15.3)	0.679^#^
Fg (g/L)	3.5 (3.1, 4.0)	1.000^*^	3.7 (3.1, 4.1)	0.026^*^	4.1 (3.4, 5.0)	< 0.001^*^	< 0.001^*^	3.6 (3.2, 4.1)	4.1 (3.3, 4.7)	**< 0.001**^#^
D-dimer (μg/L)	90.0 (59.0, 131.0)	0.045^*^	120.0 (70.0, 180.0)	0.121^*^	148.0 (89.0, 315.0)	< 0.001^*^	< 0.001^*^	94.5 (60.5, 137.5)	131.0 (85.0, 289.0)	**< 0.001**^#^
CEA (μg/L)	1.4 (0.9, 2.3)	0.662^*^	1.7 (1.3, 2.7)	0.023^*^	3.0 (1.6, 6.3)	< 0.001^*^	< 0.001^*^	1.5 (0.9, 2.3)	2.7 (1.4, 5.3)	**< 0.001**^#^
CA19-9 (U/mL)	5.9 (4.0, 10.7)	1.000^*^	7.2 (5.0, 10.9)	0.126^*^	10.5 (5.2, 23.8)	< 0.001^*^	< 0.001^*^	6.4 (4.3, 10.9)	9.0 (5.0, 19.6)	**< 0.001**^#^
TEG parameters										
R (min)	6.7 (5.8, 7.7)	1.000^*^	6.9 (6.1, 7.5)	0.051^*^	6.3 (5.5, 7.1)	0.0370^*^	0.006^*^	6.7 (5.8, 7.7)	6.2 (5.7, 7.2)	**0.013**^#^
K (min)	1.9 (1.8, 2.2)	1.000^*^	1.9 (1.7, 2.2)	< 0.001^*^	1.6 (1.3, 1.9)	< 0.001^*^	< 0.001^*^	1.9 (1.8, 2.2)	1.7 (1.4, 2.0)	**< 0.001**^#^
α-Angle (deg)	63.8 (61.1, 66.2)	1.000^*^	63.7 (61.5, 66.0)	< 0.001^*^	67.4 (63.9, 70.6)	< 0.001^*^	< 0.001^*^	63.8 (61.2, 66.2)	66.9 (62.9, 70.2)	**< 0.001**^#^
MA (mm)	59.8 (56.9, 62.9)	1.000^*^	60.8 (57.1, 63.0)	< 0.001^*^	63.5 (60.4, 67.3)	< 0.001^*^	< 0.001^*^	60.2 (56.9, 63.0)	62.9 (60.3, 66.2)	**< 0.001**^#^

The bold in the table means statistically significant (*P* < 0.05). ^a^*P*, compared between HC and BA groups, was adjusted by Bonferroni correction (before propensity score matching). ^b^*P*, compared between BA and CRC groups, was adjusted by Bonferroni correction (before propensity score matching). ^c^*P*, compared between HC and CRC groups, was adjusted by Bonferroni correction (before propensity score matching). ^d^*P*, compared across all the groups (before propensity score matching). ^e^*P*, compared between HC + BA and CRC groups (after matching). *P, tested by Kruskal–Wallis test; ^*P*, tested by chi-square test; #*P*, tested by Mann–Whitney *U*-test

*Abbreviations*: *HC* Healthy controls, *BA* Benign adenoma patients, *CRC* Colorectal cancer patients, *Hb* Hemoglobin, *PLT* Platelet, *Fg* Fibrinogen, *PT* Prothrombin time, *APTT* Activated partial thromboplastin time, *TT* Thrombin time, *CEA* Carcinoma embryonic antigen, *CA19-9* Carbohydrate antigen 19–9

**Fig. 1:**
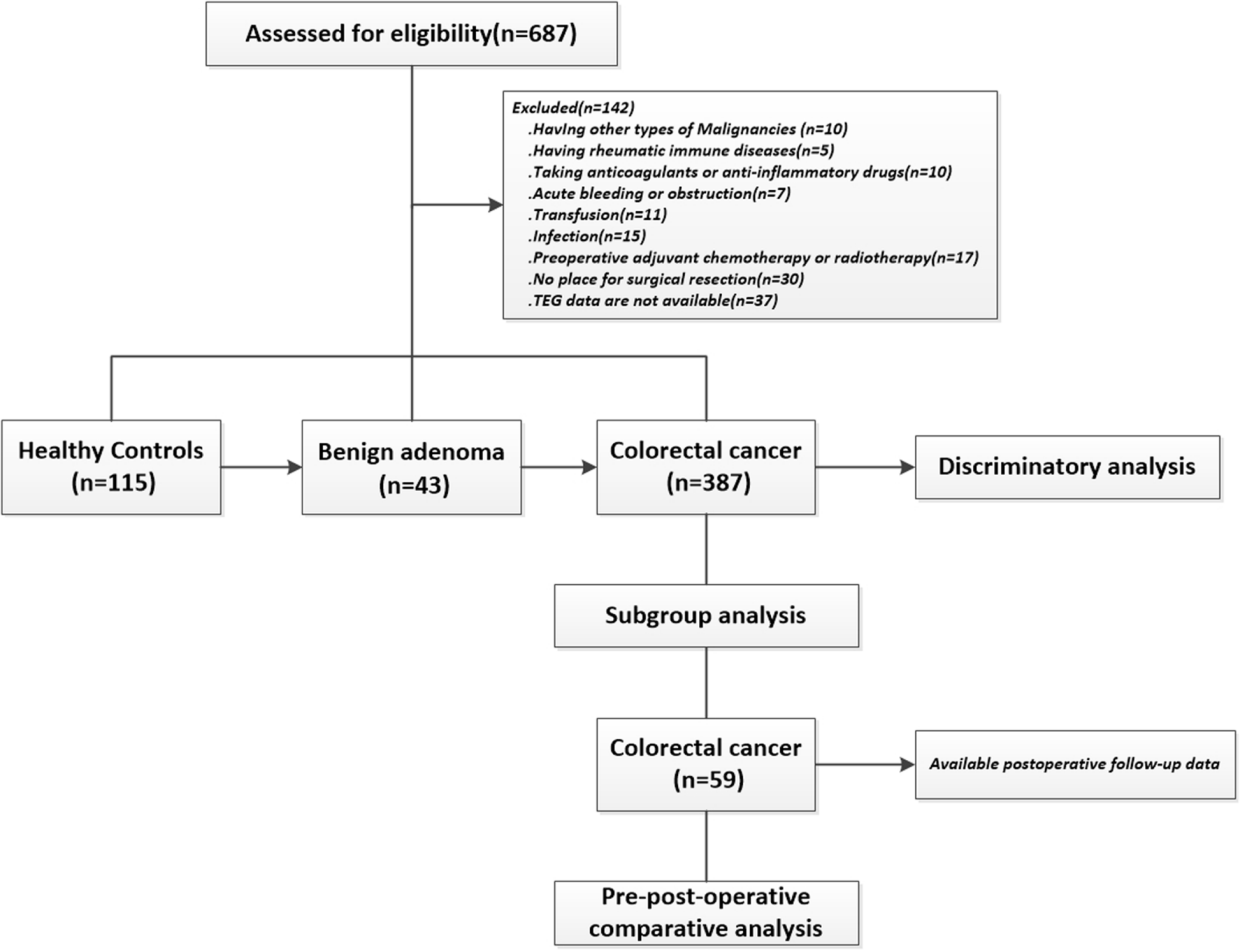
Flow chart of the study

**Fig. 2:**
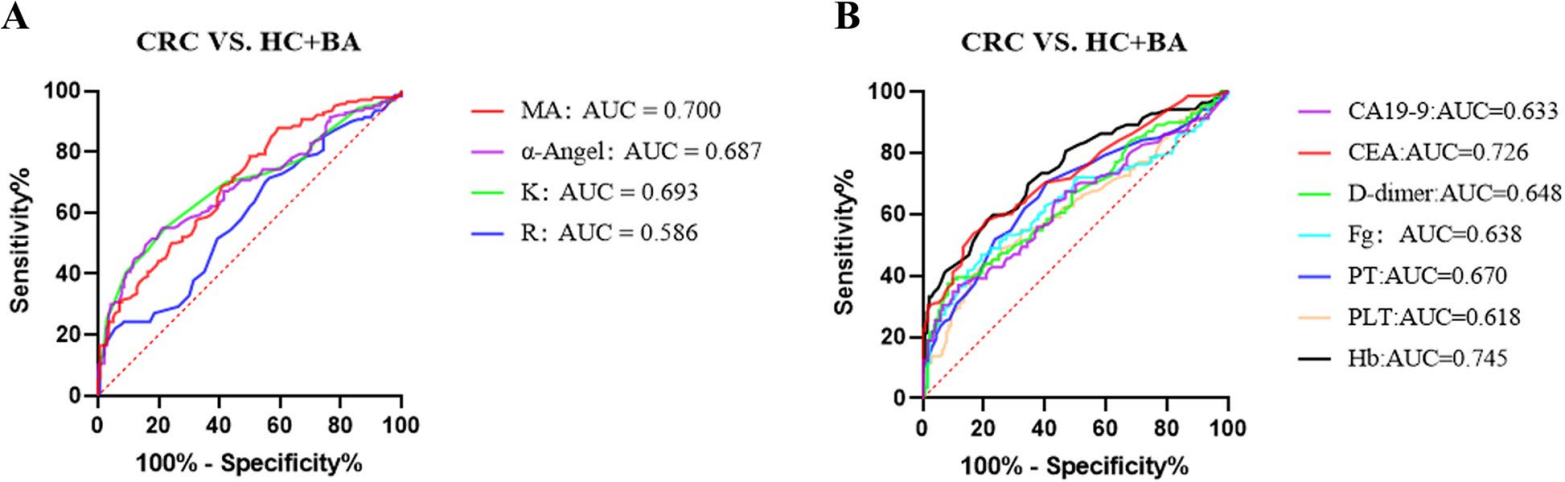
ROC curve analysis of TEG parameters and laboratory parameters for distinguishing CRC from HC + BA (after propensity score matching). **A** TEG parameters. **B** Laboratory parameters

Among the HC, BA, and CRC groups, all the TEG parameters (R, K, α-angle, and MA) reached statistical significance (Kruskal–Wallis, *P* < 0.05, Table 1). When compared in pairs, however, there was no statistical significance of TEG parameters between HC and BA groups after Bonferroni correction. The data showed that significant differences in TEG parameters still survived in the CRC group compared with the HC and BA groups, with shortened K, and higher α-angle/MA in the CRC group compared with the BA group (post hoc analysis, CRC vs. BA, *P* < 0.001, Table 1), and with shortened R/K, higher α-angle/MA in the CRC group compared with the HC group (post hoc analysis, CRC vs. HC, *P* < 0.05, Table 1). To eliminate the bias of confounding factors, we conducted PSM between HC + BA and CRC groups with a matching tolerance of 0.02. After PSM, we found that the TEG parameters had a moderate discriminative value in the identification of CRC from HC + BA. Among them, the AUC of K, α-angle, and MA were 0.693, 0.687, and 0.700, respectively. However, it is important to note that TEG parameters did not prove to be superior to traditional laboratory indicators in diagnosing CRC (Figs. 1 and 2, Table 2).

**Table 2:** The optimal cut-off values, sensitivity, specificity, PPV, NPV, and AUC for TEG parameters and laboratory parameters (distinguish CRC from HC + BA after propensity score matching)

Variable	Cutoff	Sensitivity	Specificity	PPV	NPV	AUC	95% *CI*	*P*
TEG parameters								
R (min)	5.3	22.1%	94.3%	75.0%	54.7%	0.586	0.519–0.652	0.013
K (min)	1.8	55.0%	77.9%	62.4%	65.9%	0.693	0.631–0.755	< 0.001
α-Angle (deg)	67.4	45.0%	87.9%	78.8%	61.5%	0.687	0.625–0.749	< 0.001
MA (mm)	60.0	78.6%	50.0%	61.1%	70.0%	0.700	0.639–0.760	< 0.001
Laboratory parameters								
Hb (g/L)	128.5	58.3%	78.4%	72.6%	65.3%	0.745	0.688–0.802	< 0.001
PLT (× 10^9^/L)	245.5	43.2%	80.7%	69.0%	58.5%	0.618	0.551–0.684	< 0.001
PT (s)	11.6	70.5%	59.3%	64.1%	66.9%	0.670	0.606–0.734	< 0.001
Fg (g/L)	4.2	46.8%	80.7%	70.8%	59.7%	0.638	0.572–0.704	< 0.001
D-dimer (μg/L)	198.0	37.4%	91.7%	82.5%	59.4%	0.648	0.583–0.713	< 0.001
CEA (μg/L)	2.4	58.0%	79.2%	76.2%	65.7%	0.726	0.666–0.787	< 0.001
CA19-9 (U/mL)	13.5	37.0%	88.3%	78.5%	65.7%	0.633	0.566–0.700	< 0.001

*Abbreviations*: *PPV* Positive predictive value, *NPV* Negative predictive value, *AUC* Area under the curve, *Hb* Hemoglobin, *PLT* Platelet, *Fg* Fibrinogen, *PT* Prothrombin time, *CEA* Carcinoma embryonic antigen, *CA19-9* Carbohydrate antigen 19–9

Next, we tested the association between TEG parameters and clinical parameters of CRC patients. We observed that R had no significant effect on the clinical parameters of CRC patients (*P* > 0.05, Table 3). Notably, abnormal TEG parameters, including low K (LK), high α-angle (Hα), and high MA (HMA), were strongly associated with advanced tumors. Concretely, LK was significantly associated with vascular invasion (*P* = 0.001), primary tumor status (*P* = 0.002), lymph node metastasis (*P* < 0.001), and disease stages (*P* = 0.001); Hα was significantly associated with poorly differentiation (*P* = 0.002), vascular invasion (*P* < 0.001), nerve invasion (*P* = 0.029), primary tumor status (*P* < 0.001), lymph node metastasis (*P* < 0.001), and disease stages (*P* < 0.001); and HMA was significantly associated with poorly differentiation (*P* = 0.002), vascular invasion (*P* = 0.001), nerve invasion (*P* = 0.006), primary tumor status (*P* < 0.001), lymph node metastasis (*P* < 0.001), and disease stages (*P* < 0.001) (Table 3). In addition, patients with abnormal CEA/CA19-9 also showed a higher proportion of low K (LK), high α-angle (Hα), and high MA (HMA) compared with patients with normal CEA/CA19-9 levels (*P* < 0.05, Table 2). Patients with colon cancer displayed a higher proportion of LK, Hα, and HMA than patients with rectum cancer (*P* < 0.05, Table 3). Moreover, the females were associated with Hα and HMA (*P* < 0.05, Table 3).

**Table 3:** Association of TEG parameters with clinical parameters in patients with CRC

Variable	R		*P* (*χ*^2^)	K		*P* (*χ*^2^)	α-Angle		*P* (*χ*^2^)	MA		*P* (*χ*^2^)
	LR (*n*, %)	HR (*n*, %)		LK (*n*, %)	HK (*n*, %)		Lα (*n*, %)	Hα (*n*, %)		LMA (*n*, %)	HMA (*n*, %)	
Age (years)												
≤ 65	23 (11.7)	174 (88.3)	0.719 (0.129)	13 (6.6)	184 (93.4)	0.496 (0.463)	166 (84.3)	31 (15.7)	0.273 (1.200)	174 (88.3)	23 (11.7)	0.188 (1.735)
> 65	20 (10.5)	170 (89.5)		16 (8.4)	174 (91.6)		152 (80.0)	38 (20.0)		159 (83.7)	31 (16.3)	
Sex												
Male	22 (9.5)	210 (90.5)	0.212 (1.555)	13 (5.6)	219 (94.4)	0.084 (2.985)	199 (85.8)	33 (14.2)	**0.023 (5.139)**	207 (89.2)	25 (10.8)	**0.027 (4.871)**
Female	21 (13.5)	134 (86.5)		16 (10.3)	139 (89.7)		119 (76.8)	36 (23.2)		126 (81.3)	29 (18.7)	
Preoperative CEA												
Normal	30 (11.7)	227 (88.3)	0.770 (0.085)	14 (5.4)	243 (94.6)	**0.019 (5.490)**	226 (87.9)	31 (12.1)	**< 0.001 (20.236)**	236 (91.8)	21 (8.2)	**< 0.001 (24.130)**
High	13 (10.7)	109 (89.3)		15 (12.3)	107 (87.7)		84 (68.9)	38 (31.1)		89 (73.0)	33 (27.0)	
Preoperative CA19-9												
Normal	40 (12.4)	282 (87.6)	0.116 (2.468)	21 (6.5)	302 (93.5)	**0.043 (3.869)**	272 (84.2)	51 (15.8)	**0.003 (8.057)**	291 (90.1)	32 (9.9)	**< 0.001 (32.554)**
High	3 (5.3)	54 (94.7)		8 (14.3)	48 (85.7)		38 (67.9)	18 (32.1)		34 (60.7)	22 (39.3)	
Tumor location												
Colon	28 (11.9)	208 (88.1)	0.601 (0.274)	25 (10.6)	211 (89.4)	**0.004 (8.111)**	182 (77.1)	54 (22.9)	**0.001 (11.244)**	186 (78.8)	50 (21.2)	**< 0.001 (28.065)**
Rectum	15 (10.1)	133 (89.9)		4 (2.7)	144 (97.3)		134 (90.5)	14 (9.5)		145 (98.0)	3 (2.0)	
Differentiation												
Poorly	20 (15.7)	107 (84.3)	0.085 (2.961)	15 (11.8)	112 (88.2)	0.060 (3.544)	90 (70.9)	37 (29.1)	**< 0.001 (12.860)**	97 (76.4)	30 (23.6)	**< 0.001 (12.865)**
Medium + well	22 (9.6)	207 (90.4)		14 (6.1)	215 (93.9)		198 (86.5)	31 (13.5)		207 (90.4)	22 (9.6)	
Growth pattern												
Protuberant	15 (12.7)	103 (87.3)	0.907 (0.014)	8 (6.8)	110 (93.2)	0.462 (5.40)	98 (83.1)	20 (16.9)	0.332 (0.942)	106 (89.8)	12 (10.2)	0.080 (3.074)
Ulcerative/invasive	27 (12.3)	193 (87.7)		20 (9.1)	200 (90.9)		173 (78.6)	47 (21.4)		182 (82.7)	38 (17.3)	
Vascular invasion												
Negative	26 (11.5)	200 (88.5)	0.871 (0.026)	9 (4.0)	217 (96.0)	**0.001 (10.405)**	201 (88.9)	25 (11.1)	**< 0.001 (17.446)**	206 (91.2)	20 (8.8)	**0.001 (10.854)**
Positive	17 (11.0)	138 (89.0)		20 (12.9)	135 (87.1)		112 (72.3)	43 (27.7)		123 (79.4)	32 (20.6)	
Nerve invasion												
Negative	28 (10.7)	233 (89.3)	0.612 (0.258)	18 (6.9)	243 (93.1)	0.438 (0.602)	222 (85.1)	39 (14.9)	**0.0290 (4.770)**	234 (89.7)	27 (10.3)	**0.006 (7.673)**
Positive	15 (12.5)	105 (87.5)		11(9.2)	109 (90.8)		91 (75.8)	29 (24.2)		95 (79.2)	25 (20.8)	
Tumor status												
T_1+2_	10 (8.9)	102 (91.1)	0.377 (0.779)	1 (0.9)	111 (99.1)	**0.002 (9.952)**	109 (97.3)	3 (2.7)	**< 0.001 (24.822)**	112 (100.0)	0 (0.0)	**< 0.001 (25.663)**
T_3+4_	33 (12.0)	241 (88.0)		28 (10.2)	246 (89.8)		208 (75.9)	66 (24.1)		220 (80.3)	54 (19.7)	
Nodal status												
N_0_	22 (9.4)	211 (90.6)	0.225 (1.472)	7 (3.0)	226 (97.0)	**< 0.001 (16.454)**	213 (91.4)	20 (8.6)	**< 0.001 (31.590)**	221 (94.8)	12 (5.2)	**< 0.001 (34.726)**
N_1+2_	20 (13.4)	129 (86.6)		21 (14.1)	128 (85.9)		103 (69.1)	46 (30.9)		110 (73.8)	39 (26.2)	
Metastasis status												
M_0_	35 (10.3)	306 (89.7)	0.188 (1.734)	23 (6.7)	318 (93.3)	0.206 (1.601)	292 (85.6)	49 (14.4)	**< 0.001 (22.071)**	307 (90.0)	34 (10.0)	**< 0.001 (35.834)**
M_1_	7 (17.1)	34 (82.9)		5 (12.2)	36 (87.8)		23 (56.1)	18 (43.9)		23 (56.1)	18 (43.9)	
Disease stage												
I + II	21 (9.3)	205 (90.7)	0.170 (1.881)	8 (3.5)	218 (96.5)	**< 0.001 (12.387)**	207 (91.6)	19 (8.4)	**< 0.001 (33.297)**	215 (95.1)	11 (4.9)	**< 0.001 (37.708)**
III + IV	22 (13.8)	138 (86.3)		21 (13.1)	139 (86.9)		110 (68.8)	50 (31.3)		117 (73.1)	43 (26.9)	

The bold in the table means statistically significant (*P* < 0.05). The chi-square test was used to compare TEG parameters between each variable

*Abbreviations*: *LR* Low TEG-R group, *HR* High TEG-R group, *LK* Low TEG-K group; high TEG-K group, *Lα* Low TEG-α-angle group, *Hα* High TEG-α-angle group, *LMA* Low TEG-MA group, *HMA* High TEG-MA group

**Fig. 3:**
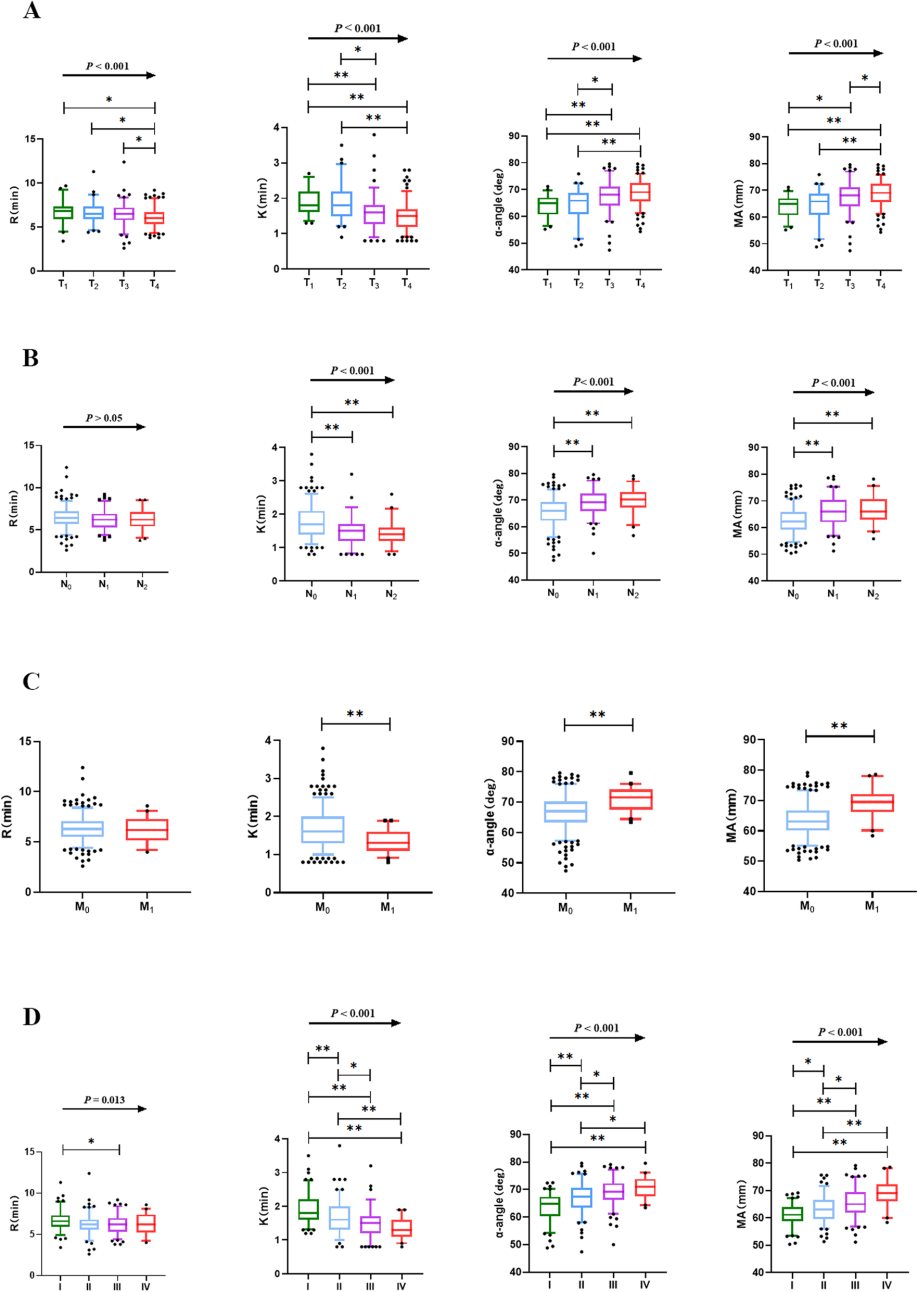
TEG parameters in CRC patients with different stages by TNM staging system. Data was described by box plot (median and 5th–95th percentile). Comparison of TEG parameters between **A** primary tumor status *T*_1_ (*n* = 50), *T*_2_ (*n* = 62), *T*_3_ (*n* = 110), and *T*_4_ (*n* = 164); **B** regional lymph node status *N*_0_ (*n* = 236), *N*_1_ (*n* = 103), and *N*_2_ (*n* = 47); **C** tumor metastasis status *M*_0_ (*n* = 345) and *M*_1_ (*n* = 41); and disease stages I (*n* = 104), II (*n* = 122), III (*n* = 119), and IV (*n* = 41) of CRC patients. *P* was tested by Jonckheere-Terpstra for an ordered difference in medians. *P was tested by the Kruskal–Wallis test and adjusted by Bonferroni correction for pairwise comparison. *Represents the *P* < 0.05. **Represents the *P* < 0.001

**Fig. 4:**
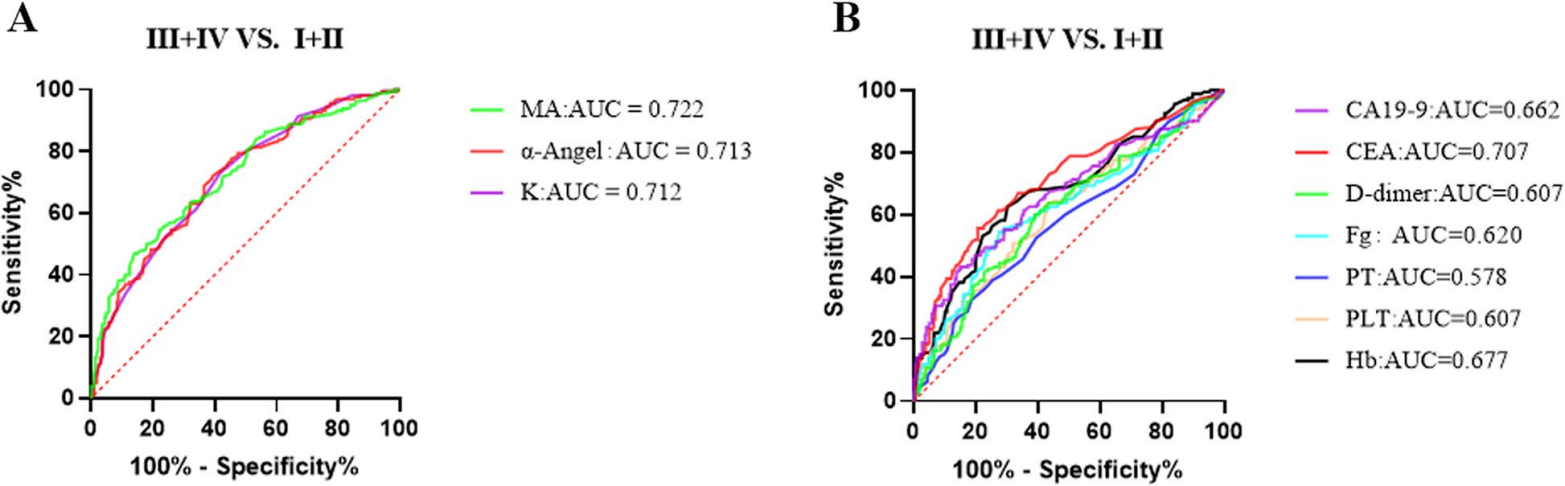
ROC curve analysis of TEG parameters and laboratory parameters for distinguishing disease stages III/IV from I/II in CRC patients. **A** TEG parameters. **B** Laboratory parameters

Further, we analyzed the relationship between TEG parameters and the TNM staging system. The Jonckheere-Terpstra test showed that K, α-angle, and MA were statistically significant for ordered difference (single increasing or decreasing) in primary tumor status (T_1_ → T_2_ → T_3_ → T_4_, *P* < 0.001, Fig. 3), lymph node status (N_0_ → N_1_ → N_2_, *P* < 0.001, Fig. 3), and disease stages (I → II → III → IV, *P* < 0.001, Fig. 3). In addition, statistics for pairwise comparisons (Kruskal–Wallis test and adjusted by Bonferroni correction) within groups were also shown in Fig. 3, showing that the more the tumor stage progressed, the more the TEG parameters were highly coagulated. Additionally, it should be highlighted that the sensitivity of K- and α-angle values in predicting advanced colorectal cancer patients, as determined by the AUC test, demonstrated a slight improvement compared to that of conventional laboratory parameters (Fig. 4, Table 4).

**Table 4:** The optimal cut-off values, sensitivity, specificity, PPV, NPV, and AUC for TEG parameters and laboratory parameters (distinguish disease stages III/IV from I/II in CRC patients)

Variable	Cutoff	Sensitivity	Specificity	PPV	NPV	AUC	95% *CI*	*P*
TEG parameters								
K (min)	1.7	73.1%	58.2%	52.9%	77.9%	0.712	0.661–0.763	< 0.001
α-Angle (deg)	67.2	71.9%	60.8%	56.4%	75.4%	0.713	0.661–0.764	< 0.001
MA (mm)	66.0	54.4%	78.0%	63.5%	70.8%	0.722	0.670–0.773	< 0.001
Laboratory parameters								
Hb (g/L)	122.5	62.5%	69.8%	58.8%	72.4%	0.677	0.621–0.731	< 0.001
PLT (× 10^9^/L)	221.5	63.1%	57.3%	51.3%	68.9%	0.607	0.549–0.665	< 0.001
PT (s)	12.5	32.5%	81.3%	55.3%	63.1%	0.578	0.520–0.636	0.009
Fg (g/L)	4.4	54.4%	72.4%	58.3%	69.3%	0.620	0.561–0.678	< 0.001
D-dimer (μg/L)	153	60.0%	60.9%	52.2%	68.5%	0.607	0.549–0.664	< 0.001
CEA (μg/L)	4.5	55.6%	79.3%	62.7%	71.0%	0.707	0.653–0.761	< 0.001
CA19-9 (U/mL)	22.1	43.1%	84.8%	62.7%	67.1%	0.662	0.605–0.719	< 0.001

*Abbreviations PPV* Positive predictive value, *NPV* Negative predictive value, *AUC* Area under the curve, *Hb* Hemoglobin, *PLT* Platelet, *Fg* Fibrinogen, *PT* Prothrombin time, *CEA* Carcinoma embryonic antigen, *CA19-9* Carbohydrate antigen 19–9

**Fig. 5:**
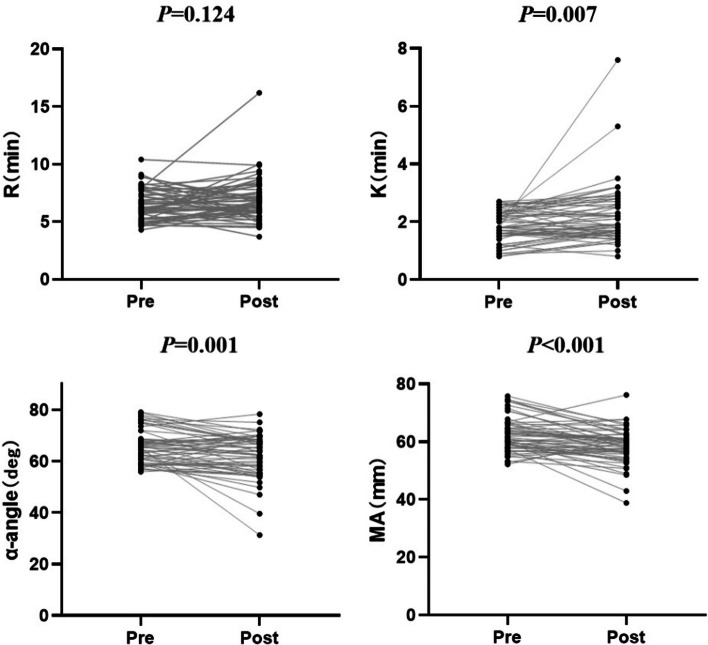
Comparison of TEG parameters between pre-operation and post-operation of CRC patients. *P* was tested by paired-samples *T*-test

During the postoperative follow-up period, only 59 CRC patients were retested for TEG (during the 3 months to 6 months after tumor excision). Surprisingly, we found a significant increase in K [1.8 (1.5, 2.3) vs. 1.9 (1.6, 2.6), *P* = 0.007] and a significant decrease in α-angle [65.3 (59.0, 68.6) vs. 63.7 (56.6, 68.5),* P* = 0.001]/MA [61.0 (58.2, 66.0) VS. 58.9 (55.8, 61.3), *P* < 0.006] after tumor surgery compared to the preoperative level (paired-samples *T*-test, Fig. 5).

## Discussion

As documented, patients with tumors were often associated with a hypercoagulable state, with no exception in colorectal cancer [18]. This hypercoagulable state, directly or indirectly mediated by tumor cells, contributes to the survival of malignant cells and the establishment of a pre-metastatic niche [19, 20]. Therefore, routine preoperative screening of CCTs in patients with tumors not only helps to predict venous thrombus embolism (VET) but also has certain diagnostic value in tumor staging and prognosis [21–24]. In this context, Zhang et al. [25] have revealed that APTT, PT, and fibrinogen were significantly associated with disease-free survival (DFS) and overall survival (OS) in patients with CRC. However, Biró et al. [26] demonstrated by multivariate regression that APTT and fibrinogen were not prognostic factors for DFS and OS in colorectal cancer patients. These controversial conclusions might be explained by the population included and the duration of follow-up but were more likely attributable to the limitation of CCTs, which reflect only one link in the clotting cascade. It is worth noting that viscoelastic tests like TEG, which utilize whole blood samples, have the capacity to provide a more comprehensive assessment of coagulation, including clotting factor function, platelet function, and fibrinolytic function.

A previous study has confirmed that TEG parameters would present a hypercoagulable condition in cancers, defined as reduced R-value, shorter K-value, broad α-angle value, and lofty MA value [4]. In the present study, more abnormal TEG parameters were displayed in CRC patients. More specifically, CRC patients have shortened K and elongated α-angle/MA compared with HC and BA patients, which was consistent with the study by de Waal and his colleagues [27]. This phenomenon indicates that CRC patients were more prone to fibrinogen and platelet activation. Besides, our data also suggested that TEG parameters (K, α-angle, and MA) could be the candidate markers in distinguishing CRC from HC + BA with moderate power. In this regard, TEG parameters could be the potential screening biomarkers in CRC patients. However, it is important to note that they did not outperform traditional indicators in this regard. Additionally, whether TEG parameters could further predict tumor progression in CRC patients remains to be resolved. To seek this answer, we attempted to analyze the correlation between TEG parameters and the clinical information and pathological results of CRC patients. Interestingly, TEG-based hypercoagulability, especially Hα and HMA, was not only associated with abnormal preoperative tumor markers (CEA and CA19-9) in patients with CRC but also with vascular and neural invasion and TNM staging. Furthermore, as the tumor stage advanced, TEG parameters showed gradual changes, characterized by a progressive decrease in K-value, while α-angle value and MA value exhibited a gradual increase. In addition, K-value and α-angle value had better sensitivity in predicting advanced colorectal cancer than CCTs. To our knowledge, this study was the first to correlate TEG parameters with pathological information about CRC and demonstrate that TEG could at least reflect the severity of CRC. Our results were also supported by previous research on other types of cancer, with lower K and higher α-angle/MA in high-risk renal cell carcinoma by Wang et al. [12] and shorter K/higher MA in advanced lung cancer by Zhou et al. [14]. The mechanism of this phenomenon can be explained by the fact that fibrinogen and platelets promote tumor development through multiple pathways.

Fibrinogen, the basic hemostatic factor, is converted into fibrin during the final phase of the clotting cascade, which not only participates in the body’s hemostatic process but also contributes to tumor growth by providing structural support and scaffolding for tumor cells to grow [28]. Activated platelets could contribute to tumor progression and metastasis through various ways: (i) by secreting various growth factors to facilitate tumor proliferation, (ii) by secreting immunosuppressive cytokines and transferring suppressor ligands to the tumor to escape from immune surveillance, and (iii) by regulating various immune cells to form microenvironments for tumor metastasis [29]. Besides, platelets and fibrin also work together: the formation of platelets-fibrin complexes protects tumor cells from shear forces in the blood flow and helps them to escape the attacks from natural killer cells [30]. Hence, plenty of clinical studies have verified that hyperthrombocytopenia and hyperfibrinemia were not only independent risk factors for CRC but also correlated with tumor progression and even tumor recurrence [31–33].

To be noted, we intriguingly found that these TEG hypercoagulability patterns in preoperative CRC patients were statistically reversed to some extent postoperatively. These observations can be interpreted as follows: After surgical resection of CRC patients, with the reduction of tumor burden, the tumor-induced hypercoagulability associated with malignancy is also alleviated, similar to the postoperative reduction of tumor markers CEA and CA19-9, suggesting that TEG parameters might be related to the prognosis of the CRC. Unfortunately, we do not have sufficient follow-up data to compare the relationship between TEG parameters and the prognosis of CRC. However, Schulick et al. [34] have reported that elevated TEG-α-angle was associated with DFS and OS among resected patients with adenocarcinoma, providing new insights into the value of TEG parameters in tumor prognosis. To better understand the prognostic value of TEG in CRC, we will conduct longitudinal follow-up studies in the future.

There were some limitations in the present study. Firstly, our study was based on a single-center and retrospective analysis, so bias in case selection cannot be avoided. Secondly, although we tried our best to make TEG parameters reflect tumor-related coagulopathy and adopted strict inclusion criteria, it was still impossible to avoid the impact of other complications in CRC patients on TEG results, just as diabetes [16] and coronary heart disease [35] could both affect TEG parameters. Thirdly, it is important to acknowledge that our study exclusively enrolled patients with confirmed pathological results. This approach led to the exclusion of a considerable number of advanced colorectal cancer (CRC) patients who were ineligible for surgery. Consequently, our study had a relatively limited number of patients with advanced cancer stages. However, based on our experience, the TEG parameters in these patients generally show a higher hypercoagulable state than in patients who can be treated with surgical excision (data not shown).

In conclusion, this study has explored the comprehensive utilization of TEG in patients with colorectal cancer. TEG parameters exhibit only moderate diagnostic value in distinguishing CRC and predicting advanced-stage tumors but are not significantly superior to traditional laboratory tests. Given the close association of TEG parameters with disease progression and surgical intervention, TEG may still hold potential as a prognostic marker for CRC patients.

## Data Availability

Data supporting the findings of this study are available from the corresponding author upon reasonable request.
